# Outcomes of cement beads and cement spacers in the treatment of bone defects associated with post-traumatic osteomyelitis

**DOI:** 10.1186/s12891-017-1614-1

**Published:** 2017-06-12

**Authors:** Xu-sheng Qiu, Yi-xin Chen, Xiao-yang Qi, Hong-fei Shi, Jun-fei Wang, Jin Xiong

**Affiliations:** Department of Orthopaedics, Nanjing University Medical School Affiliated Nanjing Drum Tower Hospital, No. 321 Zhongshan Road, Nanjing, China

**Keywords:** Cement bead, Cement spacer, Masquelet technique, Osteomyelitis, Bone defect

## Abstract

**Background:**

Cement spacers (Masquelet technique) have traditionally been used for the treatment of segmental bone defects. However, no reports have used cement spacers for the treatment of small/partial segmental bone defects associated with osteomyelitis and compared the outcomes with cement beads.

**Methods:**

We retrospectively analysed 40 patients with post-traumatic osteomyelitis of the tibia who underwent treatment, which was performed in two stages. In the first stage, thorough debridement was performed, and bone defects were filled with either antibiotic-impregnated cement beads (bead group, 18 patients) or spacers (spacer group, 22 patients). In the second stage, the cement beads or spacers were removed (for the spacer group, the induced membrane formed by the spacer was preserved) and the bone defects were filled with cancellous autografts.

**Results:**

All patients in the bead group had small/partial segmental bone defects after debridement, while 3 patients in the spacer group had large/segmental bone defects.

The mean volume of bone defects of the spacer group (40.4 cm^3^) was significantly larger than that of the bead group (32.4 cm^3^). The infection control rate (88.9%,16/18 vs 90.9%, 20/22), bone healing time (8.5 months vs 7.5 months) and complication rates (22.2%, 4/18 vs 27.2%, 6/22) were comparable between bead group and spacer group.

**Conclusion:**

The results of this study suggest that cement spacers may have an infection control rate comparable to cement beads in the treatment of bone defects associated with post-traumatic osteomyelitis. Furthermore, cement spacers could be used for the reconstruction of small/partial segmental bone defects as well as for large/segmental bone defects, whereas cement beads were not suitable for the reconstruction of large/segmental bone defects.

## Background

Osteomyelitis has been one of the most difficult and challenging problems encountered by orthopaedic surgeons [1]. Better outcomes after the treatment of osteomyelitis have been achieved with advances in modern management techniques, including early diagnosis, the use of antibiotics and aggressive surgical treatment [2]. However, as a result of the increased number of high-energy traumas and the wide use of implants, there are a higher number of infections arising from surgical procedures related to these traumatic lesions; these infections are known as post-traumatic osteomyelitis.

Successful treatment of post-traumatic osteomyelitis requires adequate management of dead space created by debridement [3]. The reconstruction of bone defects has involved a variety of techniques [4–7]. Small defects can be filled with temporarypolymethyl methacrylate (PMMA) antibiotic-impregnated beads. The beads are usually removed within several weeks and replaced with cancellous bone grafts [4]. Open bone grafting can be a useful technique when bone defects are associated with soft tissue defects and free flaps or soft tissue transfer options are limited [5]. The two most commonly used methods for large bone defects are free vascularized bone grafts [6] and the Ilizarov bone transport technique [7].

Recently, Masquelet [8] proposed a method combining morcellized cancellous bone autografts and induced membranes for the management of segmental bone defects. The Masquelet technique is a two stage procedure. The first stage includes radical debridement, soft-tissue repair by flaps if necessary, and the insertion of a PMMA cement spacer into the bone defect. The second stage is performed at least 6 to 8 weeks later. The spacer is removed and cancellous autografts are placed within the membrane. The Masquelet technique has been used for the treatment of segmental bone defects resulting from trauma, infection, aseptic necrosis, and Ewing sarcoma [9–11]. However, few studies have focused specifically on the treatment of osteomyelitis [12, 13]. Furthermore, to our knowledge, there have been no studies using this technique for the treatment of small/partial segmental bone defects [1] associated with osteomyelitis. In the present study, the Masquelet technique was used for the management of all types of bone defects associated with osteomyelitis and was compared to the use of cement beads for the treatment of bone defects associated with post-traumatic osteomyelitis.

## Methods

Between January 2011 and January 2014, 105 patients with post-traumatic osteomyelitis were identified through our database. Sixty-five patients were excluded from the study: sixty patients did not have osteomyelitis of the tibia and five patients met inclusion criteria but were lost to follow-up. The remaining forty patients with post-traumatic osteomyelitis of the tibia were retrospectively analysed for this study. From January 2011 to June 2012, 18 patients with small/partial segmental bone defects received cement beads to fill the bone defects (bead group). From July 2012 to January 2014, 22 patients with either small/partial segmental bone defects or large/segmental bone defects received cement spacers to fill the bone defects (spacer group), as we realized the potential advantages of using induced membranes for bone reconstruction. Informed consent was obtained for all patients and consent to publish was obtained for the patients whose information appears in this publication. The study was authorized by the Medical Ethics Committee of Nanjing Drum Tower Hospital and was performed in accordance with the ethical standards of the 2013 Declaration of Helsinki.

Osteomyelitis diagnoses were made using patient history, clinical signs, radiographic studies, laboratory evaluations and positive bacterial cultures. The mean duration of the disease was 6.2 months (range, 0.5-36) and 11 infections were acute (duration <3 months). The most common causative pathogenwas *Staphylococcus aureus* (Table 1), but in7 cases, no causative agent was found.

**Table 1:** Causative bacteria

	Number of cultures
Gram positive bacteria	
Staph. aureus	14
Staph. epidermidis	2
Staph. haemolyticus	1
Staph. agalactiae	1
E. faecalis	2
E. faecium	1
E. avium	1
E. gallinarum	1
Streptococci	1
Gram negative bacteria	
E.coli	3
E.cloacae	3
* E. aerogenes*	1
Klebsiella	3
Proteus	1
Pseudomonas	1
Citrobacter	1
Acinetobacter	3

### Surgical technique and postoperative care

**Fig. 1:**
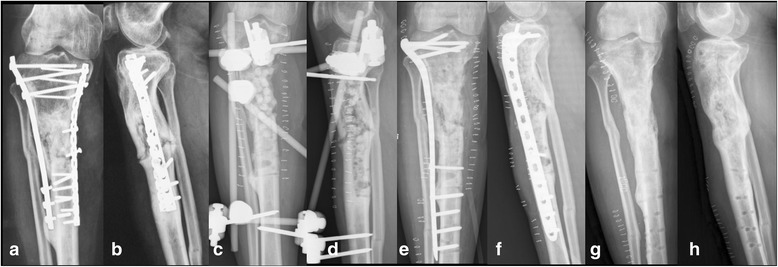
A 41-year-old male suffered from osteomyelitis of the right proximal tibia. Radiographs on admission showed loose and broken screws (**a**-**b**). The hardware was removed and a thorough debridement was performed. Partial segmental bone defect occurred after debridement. The bone defect was filled with cement beads, and the tibia was stabilized with a standard external fixator (**c**-**d**). Eighty days later, the cement beads were removed; cancellous autografts were placed within the bone defect and the external fixator was exchanged with internal fixation (**e**-**f**). The bone was healed at 11 months after the bone grafting; radiographs at 2 years follow-up showed bone union (**g**-**h**)

**Fig. 2:**
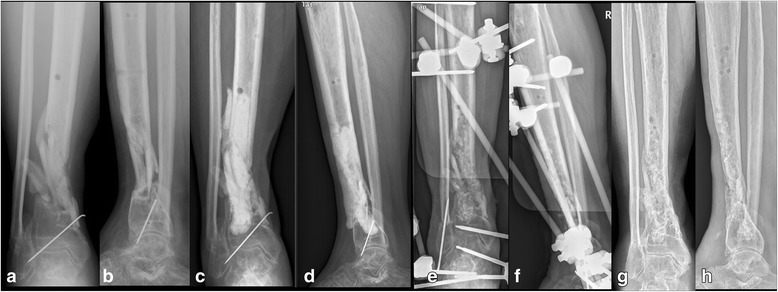
A 42-year-old male suffered osteomyelitis of the right distal tibia. X-ray films on admission showed nonunion of the tibia (**a**-**b**). A thorough debridement was performed. Segmental bone defect occurred after debridement. The bone defect was filled with a cement spacer and the lower limb was stabilized with a plaster cast (**c**-**d**). Sixty-two days later, the cement spacer was removed with preservation of the induced membrane, cancellous autografts were placed within the induced membrane, and the tibia was stabilized with a standard external fixator (**e**-**f**). The bone was healed at 8 months after the bone grafting; radiographs at 1.5 years follow-up showed bone union (**g**-**h**)

Treatment was performed in two stages: the first stage involved treating the bone infection and the second stage involved reconstructing the bone defect. Initially, any internal bone fixations were removed. After thorough soft tissue and bone debridement, the tibias of 33 patients were found to be unstable. For those patients, the lower limbs were stabilized with external fixators or plaster casts. Deep tissue samples were taken for microbiological analysis. For the bead group, the bone defect was filled with antibiotic cement beads (Fig 1); for the spacer group, the bone defect was filled with an antibiotic cement spacer (Fig 2). We used PMMA that had been premixed with gentamycin (Smith & Nephew, TN, USA) and added vancomycin to the powder before mixing the powder and liquid (2 g vancomycin per 40 g mix). The volume of the bone defect was estimated from the volume of filled cement. After the first surgical intervention, patients were initially treated intravenously with sensitive antibiotics according to previous culture results. The antibiotics were then adjusted according to the deep tissue culture results. If the culture was negative, the patient received vancomycin intravenously. Intravenous antibiotic treatment lasted until C reactive protein (CRP) and erythrocyte sedimentation rate (ESR) decreased to normal or nearly normal ranges. The patients received oral antibiotic treatment for one month. CRP levels and ESR were evaluated once again before the second stage reconstruction. Patients underwent this reconstruction stage dependent on their clinical signs and laboratory evaluations (both CRP and ESR were within normal range).

For the bead group, the second stage surgery included removing the cement beads and filling the bone defect with iliac crest cancellous bone grafts (Fig 1). For the spacer group, the second stage surgery included removing the cement spacer using an osteotome or drill, preserving the induced membrane formed by the spacer, and filling the bone defect with cancellous bone grafts within the membrane (grafts were approximately 0.3 cm −0.5 cm in size) (Fig 2). Allografts were added if there was insufficient iliac bone grafts. The external fixator was used for definitive fixation in some patients, while in other patients, the external fixator or cast was changed by internal fixation or a locking plate was used as the external fixator as the terminal treatment [14]. Intravenous antibiotic therapy was continued for 2 weeks postoperatively.

All patients were evaluated postoperatively every 1-2 months. The patients were allowed partial weight bearing after bone grafting as tolerated. Patients were allowed to bear full weight when the radiographs showed that the gaps between cancellous bone were fuzzy.

Eighteen patients needed soft tissue reconstruction. Our plastic surgery colleagues assisted on cases requiring cutaneous/fasciocutaneous flaps for soft-tissue coverage.

### Statistical analysis

Statistical analyses were conducted with SPSS version 13.0 statistical software (SPSS Inc., Chicago, Illinois). Comparison of variables between groups was performed using the independant-samples t test or Mann-Whitney U test. The frequencies of the data were statistically compared between the groups using Fisher’s exact test. A *P* value less than 0.05 was considered significant.

## Results

Patient demographics were similar between the two groups. The bead group included 18 patients (15 men and three women), whose ages ranged from 22 to 71 years and averaged 38.6 years. The spacer group included 22 patients (18 men and four women), whose ages ranged from 20 to 68 years and averaged 36.9 years.

All patients in the bead group had partial segmental bone defects [1] after debridement, while 3 patients in the spacer group had segmental bone defects. The average estimated volume of bone defects was32.4 cm^3^ (range, 15-40 cm^3^) for the bead group and 40.4 cm^3^ (range, 20-70 cm^3^) for the spacer group. The bone defects of the spacer group was significantly larger (8.1 cm^3^) than that of the bead group (*P* = 0.02). The unstable cases after thorough debridement were comparable between bead group (83.3%,15/18) and spacer group (81.8%, 18/22). There were no significant differences between bead group and spacer group for the mean time of intravenous antibiotic treatment in the first stage, the mean hospital stay in the first stage and second stage, the mean time between the first and second stage of reconstruction (*P* > 0.05) . The bead group and spacer group had comparable infection control rates (88.9%,16/18 vs 90.9%, 20/22), bone healing time (8.5 months vs 7.5 months) and complication rates (22.2%, 4/18 vs 27.2%, 6/22) (Table 2).

**Table 2:** Treatment and follow-up data

Variable	Bead Group	Spacer Group	*P*
Estimated volume of bone defects (Median (Min-Max), cm^3^)	35 (15-40)	40 (20-70)	0.02^a^
Time of intravenous antibiotic treatment (Mean ± SD, days)	15.8 ± 3.9	16.1 ± 4.1	0.84^b^
Hospital stay in the first stage (Mean ± SD, days)	23.4 ± 3.1	24.5 ± 3.3	0.28^b^
Time between the first and second stages (Mean ± SD, days)	65.7 ± 20.1	71.8 ± 20.7	0.35^b^
Hospital stay in the second stage ((Median (Min-Max), days)	10 (9-14)	9.5 (7-14)	0.40^a^
Infection control rates	88.9% (16/18)	90.9% (20/22)	1.00^c^
Bone healing time after bone grafting (Mean ± SD, months)	8.5 ± 2.1	7.5 ± 1.8	0.12^b^
Complication, No.			
Refractures	0	0	-
Superficial pin infection	4	6	1.00^c^
Pin loosening	0	0	-

^a^Mann-Whitney U test

^b^Independant-samples t test

^c^Fisher’s exact test

In the bead group, 15 tibias were shown to be unstable after a thorough debridement. External fixator were used as definitive fixation for 9 of the unstable patients, and internal fixation were used as definitive fixation for 6 patients. Four septic complications occurred after the second stage in the bead group, in two cases leading to failure of the reconstruction; another two patients had infectious complications successfully treated with prolonged antibiotic therapy as of the last follow-up. The infection control rate was 88.9% (16/18). With the exception of two failures, 16 patients achieved full weight bearing and radiographic consolidation at a mean of 8.5 months (range, 6–13) after bone grafting (Fig 1), and no refractures had occurred at a mean of 2.5 years (range, 1.5–4.3) follow-up. Superficial pin infection, observed in 4 cases, healed after intensive wound care and oral antibiotics. No pin loosening was observed. Seven patients required allografts, all flaps healed uneventfully.

In the spacer group, 18 tibias were shown to be unstable after a thorough debridement. External fixators were used as definitive fixation for all unstable patients (11 traditional external fixators, 7 with a locking plate used as the external fixator). One patient in the spacer group had failure of infection control after the initial debridement and needed further debridement and subsequent ankle fusion; another patient experienced infection recurrence 6 months after bone grafting. The infection control rate was 90.9% (20/22). One patient with a segmental bone defect had a failure of the bone reconstruction 13 months after bone grafting, which may have been due to insufficient bone grafting and unstable bone fixation. The other 19 patients achieved full weight bearing and radiographic consolidation at a mean of 7.5 months (range, 5–11) after bone grafting (Fig 2), and no refractures had occurred at a mean of 2.6 years (range, 1.5–4.5) follow-up. Superficial pin infection, observed in 6 cases, healed after intensive wound care and oral antibiotics. No pin loosening was observed. Eleven patients required allografts, all flaps healed uneventfully.

## Discussion

Successful treatment of post-traumatic osteomyelitis requires adequate management of dead space created by debridement. There are a number of options for the reconstruction of bone defects, including antibiotic-impregnated cement beads, which have been used in the treatment of chronic osteomyelitis for more than 20 years. Their use is well established: bone defects created by debridement are filled with temporary PMMA antibiotic-impregnated beads. The beads are usually removed within several weeks and replaced with cancellous bone grafts [4]. Encouraging results have been demonstrated with the use of antibiotic-impregnated beads in the treatment of chronic osteomyelitis [15–19].

The Masquelet technique is also a two-stage reconstructive procedure for the treatment of osteomyelitis defects. It was developed by AC Masquelet in the late 1970s [10]. This technique seems to have gained increasing popularity in recent years for the management of segmental bone defects. It has been used for the treatment of segmental bone defects resulting from osteomyelitis, trauma or bone tumours and encouraging results have been reported [9–11]. However, no studies have used this technique to manage small bone defects resulting from osteomyelitis. In the present study, we used the Masquelet technique to manage all types of bone defects (small/partial segmental and large/segmental bone defects) and compared the outcomes with the conventional method using cement beads.

With the use of antibiotic-impregnated beads, infection control rates in the literature range from 78% to 100% in the treatment of chronic osteomyelitis with or without systemic antibiotics [15–19]. Walenkamp et al. [18] reported on a series of 100 patients who underwent surgery using gentamicin PMMA beads with a follow-up period of 5 years. They found that the infection control rate was 78% after a single treatment period, while it was 92% after two or three treatment periods. Calhou et al. [16] evaluated the effectiveness of gentamic in PMMA beads in the treatment of osteomyelitis and found that 89.3% (25/28) of patients’ infections were successfully treated with gentamicin beads. In a retrospective study, Patzakis et al. [17] found no recurrence of infection in100% (12/12) of patients treated with gentamicin antibiotic-impregnated beads. In the present study, the infection control rate was 88.9% (16/18) in the bead group, which was similar to outcomes reported in the literature. It should be mentioned that although the final infection control rate was comparable between the bead group (16/18, 88.9%) and the spacer group (20/22, 90.9%), more septic complications were noted in the bead group (22.2%, 4/18) than in the spacer group (9.1%, 2/22); these may have been due to more internal fixations being used as definitive fixation in the bead group (33.3%, 6/18).

Few studies have specifically focused on antibiotic-impregnated spacers (Masquelet technique) in the treatment of osteomyelitis defects [12, 13]. Recently, Masquelet reviewed twelve patients with segmental bone defects resulting from infected non-unions of the tibia treated with the induced membrane technique and showed no cases of recurrent infection [13]. In the present study, the induced membrane technique was not only used for the treatment of segmental bone defects but also for the treatment of partial segmental bone defects. The overall infection control rate for the spacer group was 90.9% (20/22), and it was 89.5% (17/19) for partial segmental bone defects, which was comparable to the bead group (88.9%). It is interesting that the release of antibiotics from bone cement was influenced by the size of bone cement [20]; more antibiotics were released in the bead group than in the spacer group. However, it seems that a greater release of antibiotics did not improve the infection control rate in the bead group. There may be two reasons for this phenomenon. First, surgical debridement is critical to the success of treatment in post-traumatic osteomyelitis. Mader et al. noted that treatment failures occur if surgical debridement was inadequate, independent of the type of antibiotic used or duration of the treatment [21]. Second, although more antibiotics were released in the bead group, the local antibiotic concentration was also greater than the minimum inhibitory concentration in the spacer group. Therefore, our study indicates that antibiotic-impregnated spacers (Masquelet technique) are also an effective method for the treatment of osteomyelitis defects in terms of infection control. However, the sample size was small; a larger sample size is needed to draw definitive conclusions about infection control rates.

Removing PMMA is not as easy as removing beads, and we always spend more time removing cement spacers. However, cement spacers (Masquelet technique) have an advantage of being bone size independent [10]. They can be used for the reconstruction of small/partial segmental bone defects as well as large/segmental bone defects. The successful reconstruction of bone defects up to 25 cm in length has been reported [22]. Reconstruction is able to occur because a regular membrane is formed around the cement spacer, which prevents the resorption of cancellous bone graft and has a positive effect on consolidation of the defect [23]. One patient with a segmental bone defect had failure of bone reconstruction in the present study. This outcome may have been due to unstable bone fixation and inadequate bone grafting. Compared with spacers, beads leave an irregular membrane that is less than ideal for the containment of cancellous bone grafts [12]. Therefore, vascularized bone grafts, such as vascularized fibula flaps, are needed for the reconstruction of large/segmental bone defects when cement beads are used [24]. Vascularized bone grafts either require microsurgical anastomosis (free) or are limited by pedicle length (pedicled) and have accompanying donor site morbidity (both free and pedicled) [25, 26]. On the other hand, antibiotic-impregnated spacers (Masquelet technique) do not require specialized equipment; can be used easily by surgeons with varying experience and capability; and are applicable to patients with bone loss of epiphyseal, metaphyseal, or diaphyseal origin [27].

## Conclusion

Our study suggests that cement spacers may have an infection control rate comparable to cement beads. Cement spacers can not only be used for the reconstruction of small/partial segmental bone defects but also large/segmental bone defects. While cement beads were only suitable for the reconstruction of small/partial segmental bone defects, additional reconstruction techniques, such as vascularized fibula flaps, are needed for the reconstruction of large/segmental bone defects.

## Abbreviations

CRP: C reactive protein
ESR: Erythrocyte sedimentation rate
PMMA: Polymethyl methacrylate
